# Layered Functional Network Analysis of Gene Expression in Human Heart Failure

**DOI:** 10.1371/journal.pone.0006288

**Published:** 2009-07-24

**Authors:** Wenliang Zhu, Lei Yang, Zhimin Du

**Affiliations:** Institute of Clinical Pharmacology, The Second Affiliated Hospital of Harbin Medical University, The University Key laboratory of Hei Long Jiang Province, Heilongjiang, China; Center for Genomic Regulation, Spain

## Abstract

**Background:**

Although dilated cardiomyopathy (DCM) is a leading cause of heart failure (HF), the mechanism underlying DCM is not well understood. Previously, it has been demonstrated that an integrative analysis of gene expression and protein-protein interaction (PPI) networks can provide insights into the molecular mechanisms of various diseases. In this study we develop a systems approach by linking public available gene expression data on ischemic dilated cardiomyopathy (ICM), a main pathological form of DCM, with data from a layered PPI network. We propose that the use of a layered PPI network, as opposed to a traditional PPI network, provides unique insights into the mechanism of DCM.

**Methods:**

Four Cytoscape plugins including BionetBuilder, NetworkAnalyzer, Cerebral and GenePro were used to establish the layered PPI network, which was based upon validated subcellular protein localization data retrieved from the HRPD and Entrez Gene databases. The DAVID function annotation clustering tool was used for gene ontology (GO) analysis.

**Results:**

The assembled layered PPI network was divided into four layers: extracellular, plasma membrane, cytoplasm and nucleus. The characteristics of the gene expression pattern of the four layers were compared. In the extracellular and plasma membrane layers, there were more proteins encoded by down-regulated genes than by up-regulated genes, but in the other two layers, the opposite trend was found. GO analysis established that proteins encoded by up-regulated genes, reflecting significantly over-represented biological processes, were mainly located in the nucleus and cytoplasm layers, while proteins encoded by down-regulated genes were mainly located in the extracellular and plasma membrane layers. The PPI network analysis revealed that the Janus family tyrosine kinase-signal transducer and activator of transcription (Jak-STAT) signaling pathway might play an important role in the development of ICM and could be exploited as a therapeutic target of ICM. In addition, glycogen synthase kinase 3 beta (*GSK3B*) may also be a potential candidate target, but more evidence is required.

**Conclusion:**

This study illustrated that by incorporating subcellular localization information into a PPI network based analysis, one can derive greater insights into the mechanisms underlying ICM.

## Introduction

Heart failure (HF) is associated with significant morbidity and mortality [1]. Dilated cardiomyopathy (DCM) is a main cause for the emergence of HF, however the pathophysiological mechanisms underlying DCM are poorly understood. Over the past few years, microarray technology has been utilized extensively to reveal the global gene expression changes in DCM [2], and a fair amount of microarray expression data can be derived from public databases. However, the challenge now is how to make the most of such resources. Currently, it has been shown that an integrative analysis of gene expression and protein-protein interaction (PPI) network data is a potentially useful approach for this purpose [3], [4]. Ischemic dilated cardiomyopathy (ICM) is a main disease condition of DCM. In this paper, we report an integrative analysis linking HF gene expression and PPI network data by utilizing a publicly available ICM-related microarray data set [5].

Knowledge concerning the subcellular localization of proteins is important for providing insights about protein function and the intricate pathways that regulate biological processes at the subcellular level. This includes insights about nature of the cellular environments in which proteins interact with each other and with other molecules [6]. Consequently, a new proteomics strategy termed ‘subcellular proteomics’ has emerged in recent years [7] and has become a focus of great interest because prediction of protein subcellular location is relevant to molecular cell biology, proteomics, system biology and drug discovery [7], [8]. The importance of information on subcellular localization of proteins leads us to predict that a better understanding of diseases such as ICM, can be obtained by incorporating validated protein subcellular localization information into a traditional PPI network analysis. Such information can be retrieved from the Human Protein Reference Database (HPRD) and Entrez Gene database [9], [10]. The software Cytoscape [11] and its plugins can be used for establishing a layered PPI network by distributing nodes into differential layers according to the respective subcellular localization of the proteins.

The database of human protein atlas (HPA) is developed using antibody-based proteomic methods, which provides a comprehensive atlas for the expression and localization profiles of 48 normal human tissues and 20 different cancers [12]. Currently, more than 3500 proteins expressed in human heart can be retrieved from the HPA, and this number will certainly increase in the future. This makes HPA a very useful resource of the tissue expression profile of proteins. In order to understand the significance of the changes in gene expression of proteins in different layers the DAVID functional annotation clustering tool [13] was used to analyze the biological process terms of the differentially expressed gene in the context of the Gene Ontology (GO) [14]. The results of the GenePro [15] analysis suggest that the Janus family tyrosine kinase-signal transducer and activator of transcription (Jak-STAT) signaling pathway might play an important role in the development of ICM and could be exploited as a therapeutic target of ICM. In addition, targeting to glycogen synthase kinase 3 beta (GSK3B) may also be a potential target. Ultimately, our study illustrates that by importing subcellular localization information into a PPI network analysis, it provides unique insights into the mechanisms underlying ICM.

## Results

In this study, a portion of a microarray data set, accession number GSE1869, that included only the samples from nonfailing hearts and ICM patients, was downloaded from the Gene Expression Omnibus (GEO) [16]. Significance analysis of microarray (SAM) [17] was performed to identify differentially expressed genes. The layered PPI network was established by utilizing three Cytoscape [11] plugins, BioNetBuilder, NetworkAnalyzer and Cerebral [18]–[20]. The PPIs were established based upon integrated interactions from the DIP, BIND, Prolinks, KEGG and HPRD databases for validated cardiac myocyte proteins in the HPA and proteins encoded by genes in the gene expression data set [18]. The protein subcellular localization information was retrieved from the HPRD and Entrez Gene database [9], [10]. To distinguish between the types of proteins, each node was represented by a color coding scheme. The DAVID Functional Annotation Clustering tool [13] was applied to identify over-represented biological process in the context of GO [14]. The relationship between each of the significantly over-represented biological processes was analyzed using the Cytoscape plugin, GenePro [15].

### Layered PPI network

**Figure 1:**
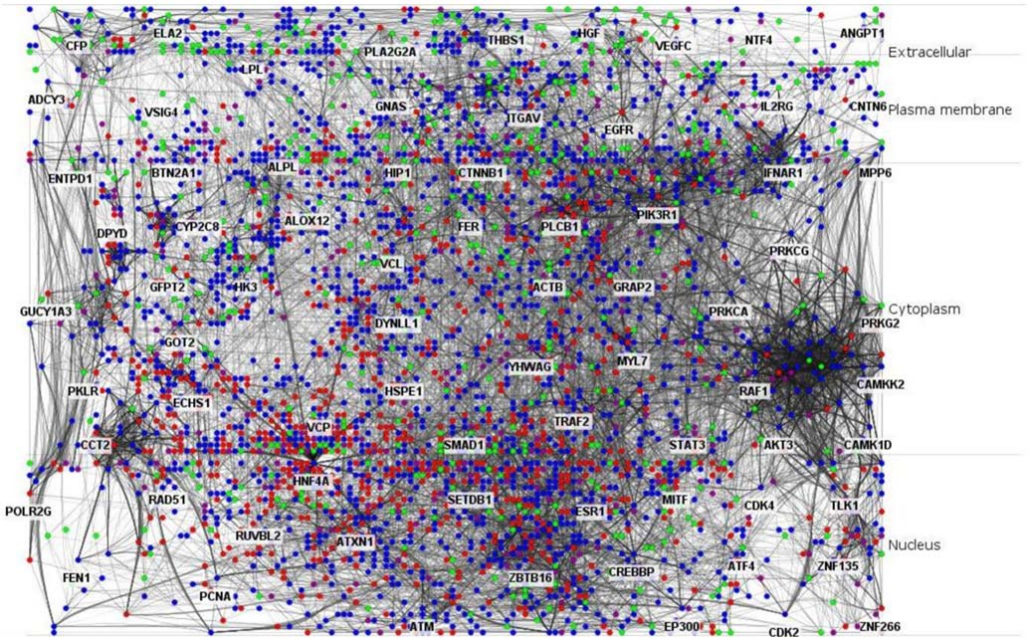
Layered PPI network. Red, green and blue nodes represent up-regulated, down-regulated and unchanged genes, respectively. Purple nodes represent other genes that have no corresponding transcripts in the data set.

In total 1888 significantly differentially expressed genes were identified using the SAM analysis, including 1114 that were up-regulated and 774 that were down-regulated, and 3549 cardiac myocytes proteins were retrieved from the HPA. Some of the proteins did not have corresponding genes in the data set. Single nodes and small components of the PPI network initially assembled were removed and only the largest component was saved as a new PPI network, which was composed of 3316 nodes and 17810 interactions. The network was comprised of 1282 proteins that were encoded by differentially expressed genes,including 776 that were up-regulated and 506 that were down-regulated; only 266 nodes remained representing proteins that were not encoded by significant differentially expressed genes (Table 1). After importing the protein subcellular localization information, the network was re-constructed and divided into four layers: extracellular, plasma membrane, cytoplasm and nucleus (Figure 1, Annex S1). In the extracellular and plasma membrane layers, the sum of proteins encoded by down-regulated genes was greater than that encoded by up-regulated genes, but in the cytoplasm and nucleus layers, this trend was reversed.

**Table 1:** Distribution of nodes.

Localization	Up-regulated	Down-regulated	Unchanged	Others	Total%
Extracellular	23	86	146	14	8.1
Plasma membrane	68	125	342	56	17.8
Cytoplasm	396	189	797	114	45.1
Nucleus	289	106	483	82	29.0
Total	776	506	1768	266	3316

### GO analysis

**Figure 2:**
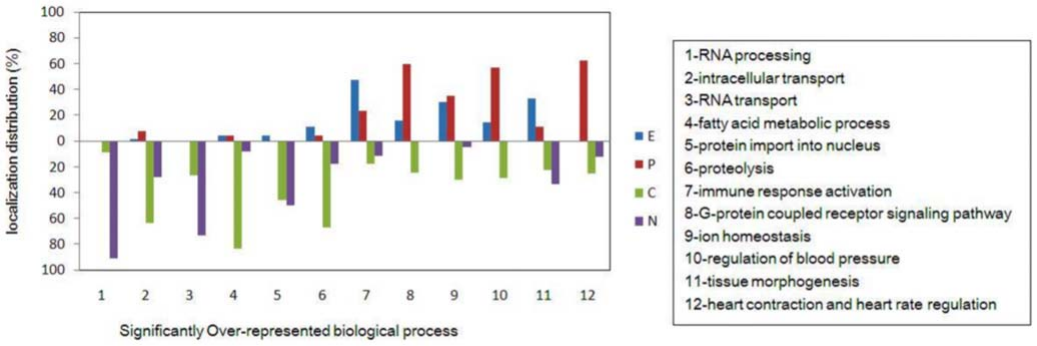
Distribution of proteins encoded by significantly over-presented genes. N: Nucleus. C: Cytoplasm. P: Plasma membrane. E: Extracellular.

To assess the layered PPI network in the context of GO, the DAVID functional annotation clustering tool was applied to identify significantly over-represented biological processes (Table 2). The protein distribution trend was also analyzed according to gene expression pattern (Figure 2). In the up-regulated gene group, most of the corresponding encoded proteins were distributed in the nucleus and cytoplasm layers. However, in the down-regulated gene group an opposite trend could be seen. Out of the six over-represented biological processes in the up-regulated gene group, there were three biological processes that were related with transport. In particular, we found that the most frequent protein and RNA transport activities were found between the nucleus and cytoplasm. In the down-regulated gene group, genes were identified that were related to biological processes that maintain normal heart cell function, including cardiac contraction and heart rate regulation, consistent with the decreased cardiac function of ICM patients found clinically. Interestingly, of the 266 genes that were not found to be differentially expressed only two were associated with over-represented biological processes. They were both related to transcriptional regulation and consequently, were not inconsistent with the above GO analysis results.

**Table 2:** GO analysis results.

Gene expression	Significantly Over-represented biological process	ES
Up-regulated	RNA processing	2.29
	intracellular transport	2.10
	RNA transport	1.63
	fatty acid metabolic process	1.63
	protein import into nucleus	1.19
	proteolysis	1.13
Down-regulated	immune response activation	2.53
	G-protein coupled receptor signaling pathway	2.08
	ion homeostasis	1.55
	regulation of blood pressure	1.08
	tissue morphogenesis	1.08
	heart contraction and heart rate regulation	1.02

GO analysis results. ES: Enrichment Score.

### GenePro analysis

**Figure 3:**
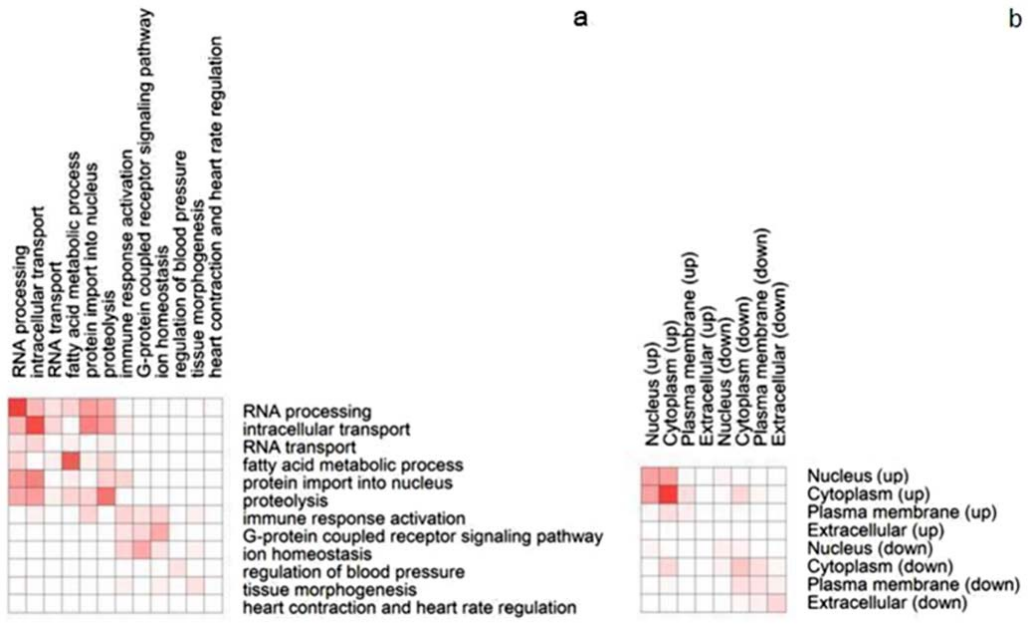
Heatmaps of the number of PPIs. The shade of red color in each grid represents the number of PPIs between the two crossed clusters. a, The number of PPIs between proteins encoded by genes of significantly over-represented GO biological processes. b, The number of PPIs between proteins in differential layers encoded by up and down regulated genes.

To assess the relationship between the significantly over-represented biological processes, the 254 encoded proteins were analyzed by using the Cytoscape plugin GenePro [15] (Annex S2). A heatmap was generated to display the number of PPIs in each cluster and between every two clusters (Figure 3a). The heatmap revealed that the greatest number of PPIs was found between clusters in which proteins were encoded by up-regulated genes. However, there were a few PPIs that were shared between the up-regulated and down-regulated biological processes. Notably, three PPIs were found that were shared between biological processes involving protein import into nucleus and immune response activation that included *JAK2* (Janus kinase 2): *STAT6* (Signal transducer and activator of transcription 6), *JAK2*:*STAT3* (Signal transducer and activator of transcription 3) and *GSK3B* (Glycogen systhase kinase 3 beta):*BCL3* (B-cell cll/lymphoma 3). The 1182 proteins encoded by up and down-regulated genes were also analyzed by utilizing GenePro (Annex S3). A heatmap was generated by distributing these proteins into 8 clusters according to their subcellular localization and gene expression pattern (Figure 3b). Once again we found that the number of PPIs identified between clusters was associated with gene expression pattern rather than protein subcellular localization. For example, there were 142 PPIs identified between proteins encoded by up-regulated genes in the nucleus and cytoplasm but only 16 PPIs identified between the proteins encoded by up and down-regulated genes in the nucleus. There were only five PPIs identified between proteins encoded by up-regulated genes in the nucleus and down-regulated genes in the cytoplasm.

## Discussion

DCM is associated with significant morbidity and mortality, and is a main cause for the emergence of HF [1]. By taking full advantage of publicly available data bases, we undertook an integrative bioinformatics approach that linked gene expression and PPI network data bases to search for mechanisms that might be implicated in DCM and ICM. The novel approach taken in our work involves the incorporation of subcellular localization information of proteins and the creation of a layered PPI network. We feel that this is a highly relevant approach because the composition and biological role of proteins vary with subcellular localization. For instance, proteins on the plasma membrane are primarily involved in cell adhesion, ion transport and cell signaling, whereas in nucleus, proteins are mainly involved in transcription and ribosomal assembly. By taking this approach we have been able to investigate the implications of differential ICM gene and protein expression in four distinct subcellular layers. We found that in patients with ICM proteins encoded by down-regulated genes were primarily found in extracellular and plasma membrane layers, whereas the up-regulated genes were found predominantly in the cytoplasm and nucleus. This finding raises the question as to why cells derived from ICM patient have weaker gene expression of proteins in extracellular and plasma membrane layers, while there is an enhancement of proteins in the other two layers. To elucidate the biological significance we used the DAVID annotation clustering tool and found a parallel reversed functional trend.

GO analysis established that proteins encoded by up-regulated genes involved in significantly over-represented biological processes were primarily located in the nucleus and cytoplasm layers, while proteins encoded by down-regulated genes were mainly located in the extracellular and plasma membrane layers. In particular, three over-presented biological processes in the up-regulated gene group were all related with the key word “transport”. It is well recognized that nuclear transport is crucial to cell function, as it is required for both gene expression and chromosomal maintenance. Thus, the present observation might suggest that there exists a significant structural modification related to nuclear transport in ICM, which might be related to the fact that abnormally large nuclei are found in cardiac myocytes from human ICM hearts. In contrast, many genes involved in maintaining normal heart functions such as cardiac contraction and heart rate regulation were down-regulated. In the context of the ischemic extracellular environment, it seems paradoxical that on one hand biological processes such as RNA processing, RNA and protein transport, and protein translation (P<0.05, not included in the functional annotation clustering report) are enhanced, whereas normal cardiac function is compromised. One possibility is that the changes observed in the cardiac myocytes from hearts of ICM patients are triggered by persistent viral infection that has been recently shown to play a pivotal role in the pathogenesis of DCM [21], [22].

To assess the relationship between the significantly over-represented biological processes, a GenePro analysis was performed. The results show that the number of PPIs between biological processes was associated with differential gene expression pattern (Figure 3a). Initially, we considered that this could be explained on the basis of protein subcellular localization, as proteins encoded by up-regulated genes were mainly distributed in the nucleus and cytoplasm layers and proteins encoded by down-regulated genes were mainly distributed in the other two layers (Figure 2). However, the GenePro analysis revealed that proteins with the same altered gene expression pattern, which might imply that the proteins could be interacting with each other, were actually located in different cell layers (Figure 3b). This suggests that over-represented biological processes might be associated with similar gene regulation patterns and could be functionally linked to each other. In other words, they may be mutually required from a functional perspective and consequently adopt the same regulation pattern. On the other hand, it is also possible that the various biological processes may actually influence each other. For instance, if one biological process was weakened, another biological process could be impaired as a consequence and visa versa. Thus, our aim was to search for such pairs of interacting proteins that were encoded by differentially regulated genes. Only three PPIs were found that included *JAK2*:*STAT6*, *JAK2*:*STAT3* and *GSK3B*:*BCL3*. It is well known that the Jak-STAT signaling pathway is broadly used by interferons and type I cytokines [23]. The role of the Jak-STAT pathway plays in the immune system makes it a prime target for therapeutic intervention [24]. Mazen et al discussed the feasibility of exploiting the protective actions of Jak-STAT in the heart therapeutically [25], but it was suggested that unbridled activation of Jak-STAT signaling could be detrimental, particularly as uncoupled from the Interleukin-6-type cytokines [25]. Consequently, it was considered that the pharmacological targeting of *JAK2* or *STAT3* may be unwise. A better approach would be to target *SOCS3* (Suppressor of cytokine signaling 3) since in the presence of *SOCS3* inhibition, sustained *STAT3* activity at a moderately heightened level may not be harmful to cardiac myocytes [25]. This requires further investigation.

There are increasing lines of evidence that *GSK3B* is an essential negative regulator of cardiac hypertrophy and that the inhibition of *GSK3B* by hypertrophic stimuli is an important mechanism contributing to the development of cardiac hypertrophy [26]. However, Shinichi et al. found that inhibition of *GSK3B* could induce well-compensated hypertrophy, inhibit apoptosis and fibrosis, and increase cardiac contractility in the failing heart of mice [27]. This suggests that the pharmaceutical targeting of *GSK3B* for the therapy of ICM in heart failure patients may also be a reasonable possibility, but more work is needed to determine the validity of this approach.

The PPI network presented here is far from being complete. While it may include some noise, false positive interactions and a few of nodes without gene expression pattern, we believe that a layered PPI network analysis can provide insight into molecular mechanisms underlying cardiac ICM and other diseases.

## Materials and Methods

### Microarray data analysis

In this study, a microarray data set downloaded from the Gene Expression Omnibus (GEO) was analyzed, accession number GSE1869 [16]. This data set, which was originally available in log scale, was derived from a study on DCM, including 6, 10 and 21 samples from nonfailing hearts, ICM and nonischemic cardiomyopathy (NICM) patients, respectively [5]. As NICM was not involved in this study, the samples of NICM hearts were discarded. The significance analysis of microarray (SAM) [17] was performed to quantity differential gene expression. If False Discovery Rate (FDR) <0.05 and Fold-change >1.2, gene expression was considered significantly different.

### Layered PPI network

Three Cytoscape [11] plugins were applied for assembling the layered PPI network. A traditional PPI network was assembled firstly by using the BioNetBuilder [18] for the validated cardiac myocyte proteins [12] and proteins encoded by genes included in the data set. The PPIs were retrieved from the DIP, BIND, Prolinks, KEGG and HPRD databases [18]. Single nodes and small components of the network were removed; only the largest component was reserved as a new network. Subsequently, duplicated edges and self-loops were also removed by using the NetworkAnalyzer [19]. The modified PPI network was composed of 3316 nodes and 17810 interactions. The protein subcellular localization information retrieved from the HPRD and Entrez Gene database [9], [10] was imported as a node attribute. Then, the Cerebral [20] was applied to redistribute nodes according to subcellular localization and the PPI network was divided into four layers, i.e. extracellular, plasma membrane, cytoplasm and nucleus. Nodes without localization information were positioned into the same layer of their closest neighbors.

In addition, nodes were labeled by different colors to indicate the types of proteins represented. Green and red nodes represented proteins encoded by down- and up-regulated genes, respectively. Blue nodes represented proteins encoded by genes not significantly differentially expressed. Purple nodes represented proteins encoded by genes whose expression pattern was not present in the data set.

### Gene Ontology analysis

To detect significantly over-represented GO biological processes, the DAVID functional annotation clustering tool [13] was used by choosing the ‘GOTERM_BP_5’ option. The Initial Group Membership and Final Group Membership were set at 2, while other settings were unchanged. The threshold value of Enrichment Score was set at 1.0 instead of 1.3 thereby avoiding the loss of some important information [13]. The gene expression of proteins represented by purple nodes was treated as not significantly different. Their corresponding genes were analyzed by the above procedure to evaluate if such treatment could influence the validity of the GO analysis. The genes included in the significantly over-represented biological process group were sorted according to the subcellular distribution of the encoded proteins.

### GenePro analysis

The Cytoscape plugin GenePro [15] was applied for assembling a PPI network in which only proteins encoded by genes of significantly over-represented GO biological processes were included. The 254 included proteins were sorted into 12 clusters representing the corresponding biological processes. A heatmap was made for displaying the number of PPIs in each cluster and between every two clusters. In addition, the 1282 proteins encoded by up-regulated or down-regulated genes were also analyzed using GenePro. They were distributed into 8 clusters according to subcellular localization and gene regulation pattern. The number of PPIs in each cluster and between every two clusters was recorded into another heatmap.

## Supporting Information

Annex S1 Layered PPI network of ICM
(0.53 MB ZIP)

Annex S2 GenePro analysis result of proteins encoded by genes of significantly over-represented GO biological processes
(0.04 MB XLS)

Annex S3 GenePro analysis result of protiens encoded by differentially regulated genes
(0.14 MB XLS)
